# Effect of diminished ovarian reserve on the outcome of fresh embryo transfer in IVF/ICSI cycles among young women: A retrospective cohort study

**DOI:** 10.1186/s12905-024-03039-6

**Published:** 2024-04-09

**Authors:** Suqin Zhu, Wenwen Jiang, Xiuhua Liao, Yan Sun, Xiaojing Chen, Beihong Zheng

**Affiliations:** 1 Center of Reproductive Medicine, Fujian Maternity and Child Health Hospital, Fujian Medical University, Fuzhou, 350001 China; 2 Fujian Maternal-Fetal Clinical Medicine Research Center, Fuzhou, 350001 China; 3 Fujian Key Laboratory of Prenatal Diagnosis and Birth Defect, Fuzhou, 350001 China; 4 Fujian Provincial Reproductive Medicine Center, Fujian Maternity and Child Health Hospital, College of Clinical Medicine for Obstetrics & Gynecology and Pediatrics, Fujian Medical University, No. 18 Daoshan Road, Fuzhou City, Fujian Province 350001 China

**Keywords:** In vitro fertilization-embryo transfer, Diminished ovarian reserve, Clinical pregnancy rate, Live birth rate, Neonatal birth weight

## Abstract

**Objective:**

This study aims to investigate the effect of diminished ovarian reserve (DOR) on the clinical outcomes and maternal and infant safety of in vitro fertilization/intracytoplasmic sperm injection (IVF/ICSI) procedures in young women aged ≤ 35 years.

**Methods:**

A retrospective cohort study was performed to analyze the clinical data of 4,203 infertile women aged ≤ 35 years who underwent fresh embryo transfer (ET) in IVF/ICSI cycles. The data were collected from their initial visits to Fujian Maternity and Child Health Hospital between January 2015 and January 2022. Based on their ovarian reserve, the participants were categorized into two groups: DOR group (*n* = 1,027) and non-DOR group (*n* = 3,176). A propensity score matching (PSM) method was employed to ensure a relatively balanced distribution of covariates. The primary outcome assessed in this study was the live birth rate, while the secondary observation indicators included rates of high-quality embryo development, blastocyst formation, clinical pregnancy, and miscarriage, along with perinatal complications, neonatal birth weight, and the incidence of low birth weight (LBW).

**Results:**

The DOR group showed notably lowered rates of blastocyst formation (59.8% vs. 64.1%), embryo implantation (29.8% vs.33.3%), clinical pregnancy (47.9% vs. 53.6%), and live birth (40.6% vs. 45.7%) compared to the non-DOR group (all *P <* 0.05). However, no statistically significant differences were observed in the high-quality embryo rate, miscarriage rate, perinatal complications, neonatal birth weight, or LBW incidence in infants between both groups (all *P* > 0.05).

**Conclusion:**

DOR has been found to reduce both clinical pregnancy and live birth rates in young females undergoing fresh ET in IVF/ICSI cycles. However, this reduction does not increase the risk of perinatal complications or LBW of infants through live birth cycles.

## Background

The ovary is a vital reproductive organ that plays a crucial role in both reproductive (oviposition and ovulation) and endocrine functions (synthesis and secretion of estrogen, progesterone, and to a lesser extent, androgen). A properly functioning ovary maintains normal menstruation and fertility, whereas diminished ovarian reserve (DOR) significantly impairs fertility [1]. DOR is characterized by ovarian insufficiency due to a decline in the quantity and/or quality of oocytes in the ovary, resulting in reduced fertility. This condition is usually accompanied by reduced anti-Müllerian hormone (AMH) levels, reduced antral follicle counting (AFC), and elevated follicle-stimulating hormone (FSH) levels [2]. DOR is frequently observed in individuals with infertility who are undergoing assisted reproduction, and its prevalence can range from 6 to 64% across different age groups [3]. DOR can be attributed to a wide array of factors, including physiological factors such as the natural decline in ovarian function associated with advanced age. Additionally, multiple pathological factors contribute to the risk of DOR, including medical factors such as a history of surgery or radiotherapy on the reproductive system, autoimmune diseases, infections, environmental influences, and psychosocial factors [4]. Individuals with DOR primarily manifest symptoms of infertility, challenges in achieving conception, elevated susceptibility to early miscarriage, recurrent miscarriage, poor responsiveness to gonadotropin (Gn) therapy, and repeated failures in embryo implantation [5, 6]. Although spontaneous pregnancy may still occur in the early stage of DOR, the monthly probability of pregnancy for the affected individuals decreases from the typical range of 20-25% in women with normal ovarian function to a range of 5-10%. Moreover, individuals with DOR are prone to spontaneous abortion and fetal chromosomal aberrations [7]. Therefore, women with DOR often encounter significant financial and emotional stress during the process of assisted conception. This study included a cohort of young (≤ 35 years) DOR patients [8]. Although these individuals displayed a lower follicle count in their ovaries, the quality of the retrieved oocytes and embryos was usually satisfactory. The outcomes after embryo transfer (ET) were commonly favorable, representing the most promising subtype of DOR patients. Before receiving assisted reproductive technology (ART) treatment, these patients typically expressed high levels of concern regarding the live birth rate, perinatal complications, and offspring health. The objective of our research is to investigate the impact of DOR on the clinical outcomes and maternal and infant safety through IVF/ICSI procedures in young women aged ≤ 35 years.

## Methods

### Subjects

A retrospective cohort study was performed on 4,203 females (aged ≤ 35 years) who presented with infertility and underwent their initial IVF/ICSI cycle between January 2015 and January 2022. These participants were classified into the DOR and non-DOR groups based on their ovarian reserve status. Patients diagnosed with DOR met at least two of the following three specific criteria: (1) AMH < 1.2 ng/mL, (2) AFC < 7 on days 2–4 of the menstrual cycle, (3) basal serum FSH > 10 U/L [9]. Females with normal ovarian reserve function were classified in the non-DOR group. The inclusion criteria for this study were as follows: (1) female patients aged ≤ 35 years, (2) patients who received IVF/ICSI-ET for the first time, (3) those who used fresh ET, (4) those with complete data. The exclusion criteria were: (1) polycystic ovary syndrome, (2) recurrent miscarriage, (3) uterine malformation, (4) chromosomal abnormalities in either spouse, (5) autoimmune diseases, (6) hypertension, (7) diabetes mellitus. Approval for this investigation was granted by the Ethics Committee of Provincial Maternal and Child Health Hospital of Fujian (2021KYRD09036). All patients provided informed consent.

### Controlled superovulation procedures

Patients who underwent the long protocol during the follicular phase were administered subcutaneous injections of gonadotropin (Gn)-releasing hormone agonist (GnRH-a) (long-acting Diphereline, 3.75 mg; Ipsen Pharma Biotech, France) on the third day of the menstrual cycle. In addition, they received Gn and recombinant FSH (r-FSH, Gonal-F, 450 IU; Merck, Germany) at a daily dose ranging from 100 to 225 IU. In cases where LH < 1.0 U/L during ovulation, supplementation with recombinant luteinizing hormone (r-LH, Luveris, 75 IU; Merck, Germany) was provided. In the antagonist protocol, patients were given daily subcutaneous injections of r-FSH from the second to the fourth day of their menstrual cycle. Subsequently, they received daily subcutaneous injections of GnRH-A (cetrorelix acetate for injection, Cetrotide, 0.25 mg; Merck, Germany) at a dominant follicle diameter of > 12 mm or blood estradiol levels of 200–400 ng/L. When the desired follicle size criteria were met, such as one dominant follicle reaching 18 mm in diameter, two dominant follicles reaching 17 mm in diameter, or three dominant follicles reaching 16 mm in diameter, patients were given intramuscular or subcutaneous injections of 10,000 IU of human chorionic gonadotropin (hCG, 2000 IU; Livzon Pharmaceutical Group, Zhuhai, China), 0.25 mg trigger of recombinant hCG (rhCG, Ovidel, 250 µg; Merck, Germany) or a combination of hCG 2000 IU and GnRH-a 0.2 mg (short-acting Dabigat, 0.1 mg, Pfizer, Switzerland). The selection of hCG or rhCG as the trigger was applied in the long-acting regimen, while the single trigger of hCG or rhCG, or a double trigger of GnRH-a + hCG was chosen in the antagonist regimen.

### Embryo culture

Oocyte retrieval was conducted 36–37 h following the administration of the HCG injection, with the procedure guided by transvaginal ultrasound. The IVF/ICSI was performed in the embryology laboratory following the standard operating procedures of our center. The fertilization status was assessed 16–20 h after fertilization, followed by morphological evaluation of embryos. High-quality embryos were identified based on specific criteria, including two pronuclei (2PN) origin, cell count of 6–10 on day 3 (D3), < 25% fragmentation, symmetric blastomeres, and absence of multinucleated blastomeres [10]. The Gardner and Schoolcraft scoring system was used to evaluate the blastocytes [11]. A vitrification kit (Kato, Japan) was used to cryopreserve and freeze the remaining embryos.

### Fresh ET and luteal support

According to the number of blastomes, blastomes uniformity and fragmentation ratio, the embryos were divided into 4 grades. (1) Grade I embryos: 6 ∼ 9blastomes, uniform size, fragmentation degree 0 ∼ 5%; (2) Grade II embryos: the size is basically uniform, and the degree of fragmentation is 10–25%; (3) Grade III embryos: uneven blastomere, fragmentation degree of 25% ∼ 50%; (4) Grade IV embryos: blastomere is very uneven, fragmentation degree > 50%. The embryos with grade I to II morphological rating are the high quality. The development of blastocyst was observed on the 5th and 6th day of fertilization, respectively. The observation indexes included the size of blastocyst cavity, inner cell mass and trophoblast cells. The blastocysts rated as 4, 5, 6(AA, AB, BA, BB) belong to the high quality blastocysts. The vast majority of young patients with DOR received high-quality cleavage stage embryo transfer in fresh cycles. However, when the patient has no high-quality cleavage stage embryo formation, blastocyst culture was performed on the available embryos, the formation of transplantable blastocyst transfer is performed. The remaining un-transferred embryos were cryopreserved using vitrification, with the informed consent of the couples. For fresh cycle transfers, luteal support was initiated on the day of oocyte retrieval. This procedure involved daily vaginal applications of sustained-release progesterone gel (Crinone, 90 mg, Merck Serono, Germany), along with oral administration of dydrogesterone (Dydrogesterone, 10 mg/tablet, Abbott, the Netherlands) at a dose of 10 mg twice a day.

### Determination of pregnancy outcome

The serum hCG was detected 14 days after the ET procedure. A biochemical pregnancy diagnosis was made when blood hCG levels were ≥ 25 IU/L. For cases of biochemical pregnancy, an ultrasound examination was conducted 28 days after transfer to confirm clinical pregnancy, indicated by the presence of a gestational sac. Embryo implantation rate is defined as the percentage of the number of cysts shown on ultrasound to the number of embryos transferred at the cleavage stage. Ectopic pregnancy was diagnosed when the gestational sac was found in locations such as the fallopian tube, pelvic cavity, cervix, cesarean scar, or abdominal cavity. In this study, miscarriage was defined as pregnancy termination before 28 weeks of gestation. Preterm delivery was defined as a live birth (at < 37 weeks of gestation). Additionally, this study classified low birth weight (LBW) as a birth weight < 2500 g.

### Main outcome measures

The main variables are listed as follows: (1) Patient demographics: age, infertility duration, body mass index (BMI), estradiol levels, basal FSH, AFC, AMH, LH, primary/secondary infertility, cause of infertility, type of fertilization (IVF/ICSI), number of embryos transferred, type of embryos transferred (embryos at cleavage stage/blastocysts); (2) Ovulation induction and response: initiation dose of Gn, duration of Gn use, total Gn use, estradiol levels on the hCG day, progesterone levels on the hCG day, endometrial thickness on the hCG day; (3) Embryology parameters: number of oocytes retrieved, maturation rate of metaphase II (MII) oocytes, cleavage rate, high-quality embryo rate, blastocyst formation rate; (4) Pregnancy outcomes: implantation rate, clinical pregnancy, multiple pregnancy, miscarriage, ectopic pregnancy, and live birth; (5) Perinatal complications: preterm delivery rate, incidence of gestational hypertension, incidence of gestational diabetes, occurrence of premature rupture of membranes, cesarean section rate; 6.Neonatal birth weight, incidence of LBW.

The primary observation indicator was the live birth rate, and the secondary observation indicators included the rates of the high-quality embryo, blastocyst formation, clinical pregnancy, and miscarriage, along with perinatal complications, neonatal birth weight, and incidence of LBW.

### Statistical analysis

Statistical analysis was conducted utilizing SPSS 26.0 (IBM Corp., Armonk, NY, USA). Before comparing two groups of samples, the Shapiro-Wilk test was employed to determine whether the samples exhibited a normal distribution. In the presence of normal distribution, the mean ± standard deviation was used to describe the statistical results, and the independent sample *t*-test was utilized to compare the two groups. Categorical variables were expressed as % (n/N). For each variable, appropriate tests were adopted to assess the statistical differences between the groups, including the Mann-Whitney U rank sum test for continuous variables and the chi-square test for categorical variables. Statistical significance was determined at *P <* 0.05. Propensity score matching (PSM) was utilized to address any imbalances in baseline characteristics and sample size between the groups. Propensity scores were calculated based on the characteristics of potential confounding variables that were considered to influence ART outcomes. The potential confounding variables included BMI, female age, male age, type, cause and duration of infertility, fertilization method (IVF/ICSI), number of embryos transferred, and type of embryos transferred. Subjects were 1:1 matched using nearest neighbor matching without replacement, with a random order and a caliper value of 0.05.

## Results

### Baseline characteristics

**Fig. 1:**
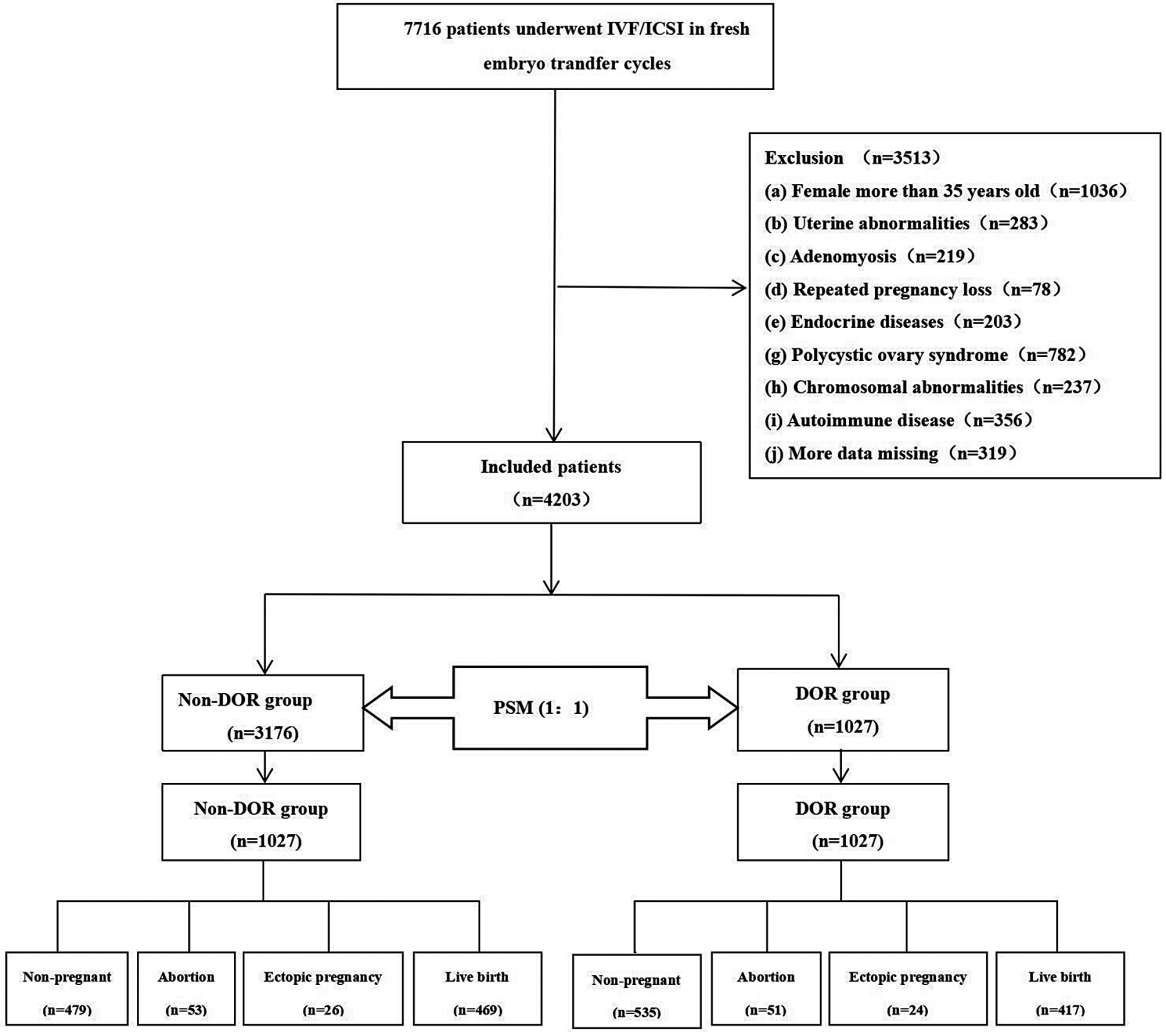
Flow chart of the study. IVF: in vitro fertilization; ICSI: intracytoplasmic sperm injection; ET: embryo transferred; DOR: diminished ovarian reserve; PSM: propensityscore matching

**Fig. 2:**
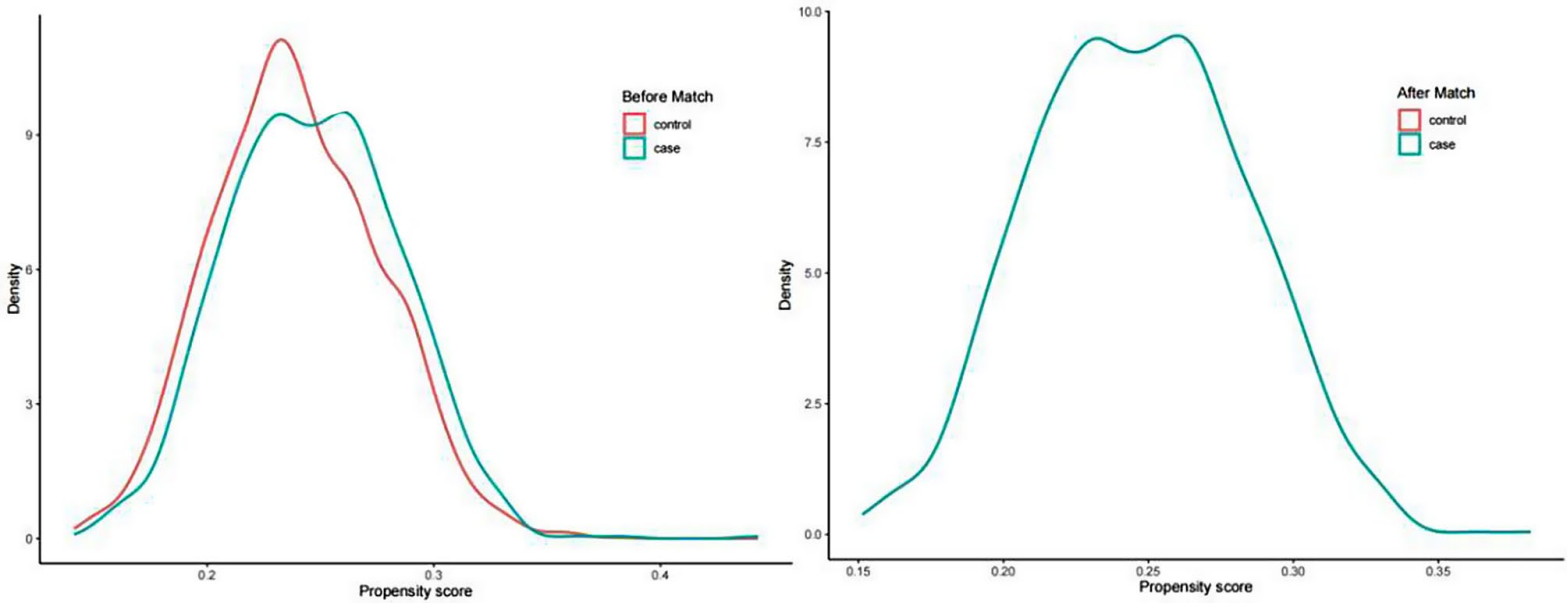
Propensity scores before and after matching

Initially, data from 7,716 patients undergoing their first fresh ET cycle with IVF/ICSI-assisted conception were screened for this study. In total, 4,203 patients were eventually included as per the inclusion and exclusion criteria and allocated into the DOR group (*n* = 1,027) and the non-DOR group (*n* = 3,176; control group). The above two groups were propensity score matched to eradicate imbalance in baseline characteristics and sample size between the groups. Significant differences were observed in the age of males and females, duration of infertility, and type of embryos transferred before matching (*P* < 0.05, Table 1). PSM (1:1) was subsequently conducted to eradicate any disparities in baseline characteristics (Fig. 1). There were 1,027 cases in each of the two groups after the intersection, and no notable differences were observed between the intersected cohorts except for indicators of ovarian reserve, such as AMH, basal FSH, and AFC (Table 1). Propensity scores were visualized, and the density curves of the scores were closely matched after matching (Fig. 2).

**Table 1:** Baseline characteristics of participants before and after matching

Variable	Unmatched				Matched				
	Control (*n* = 3176)	DOR (*n* = 1027)	t/χ2	p-value	Control (*n* = 1027)	DOR (*n* = 1027)	t/χ2		p-value
Age, y									
Male	30.09 ± 2.87	31.07 ± 2.83	-9.632	0.000*	31.04 ± 2.86	31.07 ± 2.83	-0.263	0.792	
Female	32.43 ± 3.90	33.10 ± 3.99	-4.791	0.000*	33.12 ± 3.99	33.10 ± 3.99	0.099	0.921	
Infertility duration, y	3.43 ± 2.59	3.68 ± 2.38	-2.993	0.003*	3.74 ± 2.31	3.68 ± 2.38	0.580	0.562	
BMI, kg/m^2^	21.58 ± 2.91	21.54 ± 2.93	0.312	0.755	21.62 ± 2.93	21.54 ± 2.93	0.342	0.733	
TSH, mIU/L	2.24 ± 1.03	2.27 ± 1.09	-0.777	0.437	2.26 ± 1.02	2.27 ± 1.09	-0.215	0.830	
Baseline FSH, IU/mL	6.15 ± 1.72	8.12 ± 2.06	-27.685	0.000*	6.33 ± 1.51	8.12 ± 2.06	-22.459	0.000*	
Baseline LH, IU/mL	3.85 ± 2.03	3.67 ± 2.09	2.452	0.014*	3.64 ± 2.12	3.67 ± 2.09	-0.323	0.747	
Baseline estradiol, pg/mL	42.65 ± 31.16	44.72 ± 38.23	-1.574	0.116	43.93 ± 35.24	44.72 ± 38.23	-0.487	0.626	
AMH, ng/mL	3.41 ± 1.98	1.03 ± 0.62	59.339	0.000*	3.24 ± 1.73	1.03 ± 0.62	38.538	0.000*	
AFC	10.73 ± 3.41	5.63 ± 2.14	56.595	0.000*	10.35 ± 3.52	5.63 ± 2.14	36.719	0.000*	
Infertility type, % (n)			0.105	0.746			0.502	0.479	
Primary	52.49 (1667)	53.07 (545)			54.63 (561)	53.07 (545)			
Secondary	47.51 (1509)	46.93( 482)			45.37( 466)	46.93( 482)			
Infertility causes, %(n)			1.094	0.895			2.145	0.709	
Tubal factor	42.98(1365)	42.65(438)			41.57(427)	42.65(438)			
Male factor	23.77(755)	23.66(243)			24.54(252)	23.66(243)			
Unexplained	15.62(496)	14.80(152)			13.92(143)	14.80(152)			
Endometriosis	10.20(324)	11.10(114)			10.61(109)	11.10(114)			
Others	7.43(236)	7.79(80)			9.35(96)	7.79(80)			
Fertilization rate, % (n)			2.933	0.087			1.649	0.199	
IVF	70.94 (2253)	73.71( 757)			71.18(731)	73.71( 757)			
ICSI	29.06(923)	26.29 (270)			28.82(296)	26.29(270)			
No. of embryos transferred	1.71 ± 0.45	1.73 ± 0.47	-0.985	0.325	1.71 ± 0.46	1.73 ± 0.47	-1.205	0.228	
Cleavage embryo or blastocyst transfer, % (n)			5.488	0.019*			0.912	0.340	
Cleavage embryo	93.99(2985)	95.91(985)			95.03(976)	95.91(985)			
Blastocyst	6.01(191)	4.09(42)			4.97(51)	4.09(42)			

*Note* Categorical variables are presented as % (number). Continuous variables are presented as the mean ± SD. SD: standard deviation. *P < 0.05 was considered statistically significant. PSM: propensity score matching; BMI: body mass index; TSH: thyroid stimulating hormone; FSH: folliclestimulating hormone; LH: luteinizing hormone; AMH: antiMüllerian hormone; AFC: antral follicle count

### Ovulation induction, response and embryo laboratory outcomes

No notable differences were observed in the duration of Gn use, endometrial thickness on the hCG day, maturation rate of MII oocytes, cleavage rate, or high-quality embryo rate between the two groups before and after matching (all *P >* 0.05, Table 2). The initiation dose of Gn (180.9 ± 51.6 vs.173.5 ± 51.2) and total Gn use (2919.0 ± 971.2 vs.2762.1 ± 894.7) were significantly elevated in the DOR group after PSM compared to the non-DOR group (all *P* < 0.05). Whereas, the estradiol levels on the hCG day (2270.5 ± 1345.0 vs.2789.7 ± 1421.5), serum progesterone levels on the hCG day (0.6 ± 0.4 vs.0.7 ± 0.3), the number of oocytes retrieved (4.5 ± 2.1 vs.7.8 ± 3.7), and blastocyst formation rate (59.8% vs.64.1%) were significantly reduced in the DOR group compared to the non-DOR group (all *P* < 0.05, Table 2).

**Table 2:** Ovulation induction, response and embryo laboratory outcomes before and after matching

Variable	Unmatched				Matched			
	Control (*n* = 3176)	DOR (*n* = 1027)	t/χ2	p-value	Control (*n* = 1027)	DOR (*n* = 1027)	t/χ2	p-value
COH protocol, % (n)			12.130	0.000*			1.217	0.270
GnRH-agonist	74.02(2351)	68.45(703)			70.69(726)	68.45(703)		
GnRH-antagonist	26.98(825)	31.55(324)			29.31(301)	31.55(324)		
Gn starting dosage, IU	170.20 ± 50.12	180.93 ± 51.62	-5.829	0.000*	173.53 ± 51.23	180.93 ± 51.62	-3.261	0.001*
Gn duration, d	12.53 ± 2.02	12.61 ± 2.16	-0.358	0.721	12.54 ± 2.08	12.61 ± 2.16	-0.748	0.454
Gn dosage, IU	2746.45 ± 886.64	2918.99 ± 971.18	-5.053	0.000*	2762.12 ± 894.73	2918.99 ± 971.18	-3.807	0.000*
Estradiol on HCG day, pg/mL	2824.25 ± 1417.82	2270.52 ± 1344.97	11.316	0.000*	2789.69 ± 1421.48	2270.52 ± 1344.97	7.653	0.000*
Progesterone on HCG day, ng/mL	0.75 ± 0.36	0.69 ± 0.35	4.525	0.000*	0.72 ± 0.34	0.64 ± 0.35	1.197	0.000*
Endometrium thickness on HCG day, mm	11.63 ± 2.90	11.51 ± 3.74	1.079	0.281	11.58 ± 2.97	11.51 ± 3.74	0.470	0.639
No.of oocytes retrieved	10.65 ± 3.92	4.52 ± 2.07	64.578	0.000*	7.82 ± 3.71	4.52 ± 2.07	35.781	0.000*
Mature oocyte rate, % (n/N)	56.35(19,062/33,825)	55.67(2588/4649)	0.784	0.376	54.923.14(4413/8035)	55.67(2588/4649)	0.662	0.416
Normal cleavage rate, % (n/N)	94.91(25,719/27,097)	95.15(3688/3876)	0.390	0.532	94.70(6194/6541)	95.15(3688/3876)	1.033	0.310
High-qualityembryo rate, % (n/N)	60.55(15,573/25,719)	60.17(2219/3688)	0.197	0.657	60.20(3729/6194)	60.17(2219/3688)	0.001	0.972
Blastocyst formation rate, % (n/N)	66.28(8204/12,378)	59.8(590/986)	16.838	0.000*	64.13(1647/2568)	59.8(590/986)	5.642	0.018*

*Note* Categorical variables are presented as % (number) or % (number/Number). Continuous variables are presented as the mean ± SD. SD: standard deviation. Statistical significance is determined at *P < 0.05. Gn: gonadotropin, hCG: human chorionic gonadotropin

### Clinical outcomes

No statistically significant differences were noted in the rates of multiple pregnancy, ectopic pregnancy, and miscarriage in fresh ET cycles between the two groups before and after PSM (all *P* > 0.05). After PSM, the live birth rate (40.6% vs. 45.7%), clinical pregnancy rate (47.9% vs. 53.6%), and embryo implantation rate (29.8% vs. 33.3%) were significantly reduced in the DOR group compared to the non-DOR group (all *P <* 0.05, Table 3).

**Table 3:** Clinical outcomes before and after matching

Variable	Unmatched				Matched			
	Control(*n* = 3176)	DOR(*n* = 1027)	χ2	p-value	Control(*n* = 1027)	DOR(*n* = 1027)	χ2	p-value
Implantation rate, % (n/N)	34.32(1864/5431)	29.84(530/1776)	12.104	0.001*	33.33(587/1761)	29.84(530/1776)	4.987	0.026*
Clinical pregnancy rate, % (n/N)	53.93(1713/3176)	47.91(492/1027)	60.116	0.000*	53.35(548/1027)	47.91(492/1027)	6.108	0.013*
Multiple pregnancy rate, % (n/N)	22.53(386/1713)	21.74(107/492)	0.136	0.712	21.90(120/548)	21.74(107/492)	0.003	0.953
Abortion rate, % (n/N)	9.51(163/1713)	10.37(51/492)	0.315	0.574	9.67(53/548)	10.37(51/492)	0.139	0.709
Ectopic pregnancy rate, % (n/N)	4.90(84/1713)	4.88(24/492)	0.001	0.981	4.74(26/548)	4.88(24/492)	0.10	0.978
Live birth rate, % (n/N)	46.16(1466/3176)	40.60(417/1027)	9.684	0.002*	45.67(469/1027)	40.60(417/1027)	5.367	0.012*

*Note* Categorical variables are presented as % (number/Number). Continuous variables are presented as the mean ± SD.SD: standard deviation. Statistical significance is determined at *P* < 0.05

### Pregnancy complications and neonatal outcomes

Before PSM, there were 985 fresh cycle live births in the non-DOR group and 380 fresh cycle live births in the DOR group. However, there were 293 fresh cycle live births in the non-DOR group and 275 fresh cycle live births in the DOR group after PSM. No significant differences were found in the preterm delivery rate and complications, such as gestational hypertension, gestational diabetes, premature rupture of membranes, and cesarean section, between the two groups before and after PSM (all *P >* 0.05, Table 4). The birth weight in the DOR group was (3,276.27 ± 491.24) g, and that in the non-DOR group was (3,301.34 ± 487.43) g after PSM. There was no statistically significant difference in the proportion of LBW between the DOR group and the non-DOR group (16.1% vs. 15.1%) (*P* > 0.05, Table 4).

**Table 4:** Obstetrical and neonatal outcomes before and after matching

Variable	Unmatched				matched			
	Control(*n* = 1466)	DOR(*n* = 417)	t/χ2	p-value	Control(*n* = 469)	DOR(*n* = 417)	t/χ2	p-value
Premature birth rate,% (no)	13.43(197)	11.75(49)	0.814	0.367	12.15(57)	11.75(49)	0.034	0.854
Gestational hypertension,% (no)	4.23(62)	4.56(19)	0.084	0.771	4.69(22)	4.56(19)	0.009	0.924
Gestational diabetes,% (no)	13.57(199)	14.63(61)	0.303	0.582	13.85(65)	14.63(61)	0.107	0.744
PROM,% (no)	20.60(302)	22.06(92)	0.419	0.517	21.75(102)	22.06(92)	0.013	0.910
Cesarean section,% (no)	35.27(517)	37.89(158)	0.972	0.324	36.46(171)	37.89(158)	0.193	0.660
Low birth weight rate,% (no)	15.08(221)	16.07(67)	0.247	0.619	15.14(71)	16.07(67)	0.145	0.704
Birth weight, g	3297.86 ± 495.86	3276.27 ± 491.24	0.752	0.452	3301.3 ± 487.43	3276.27 ± 491.24	0.309	0.757

*Note* Categorical variables are presented as % (number). Continuous variables are presented as the mean ± SD. SD: standard deviation. Statistical significance is determined at *P* < 0.05

## Discussion

The diagnosis of DOR was initially not standardized in the field of reproductive research and was often referred to as “ovarian hyporesponsiveness.” In 2015, the Patient-Oriented Strategies Encompassing Individualized Oocyte Number (POSEIDON) criteria were introduced, which classify DOR into four subgroups based on age, ovarian reserve, and previous ovarian responsiveness. These criteria also propose specific clinical treatment options and prognostic analysis for different subgroups [8]. In 2022, Reproductive Endocrinology & Fertility Preservation Section of Chinese Society on Fertility Preservation published the Expert Group of Consensus on Clinical Diagnosis & Management of Diminished Ovarian Reserve. Therefore, in our study, patients diagnosed with DOR met at least two of the following three specific criteria: (1) AMH < 1.2 ng/mL, (2) AFC < 7 on days 2–4 of the menstrual cycle, (3) basal serum FSH > 10 U/L [9]. Different inclusion criteria of the studies will lead to different results of the studies. DOR can be caused by various factors, including environmental influences, infections, autoimmune diseases, metabolic disorders, smoking, medical factors (such as chemotherapy, radiotherapy, and ovarian surgery), and genetic abnormalities [12]. Individuals with DOR often present difficulties in achieving fertility, challenges in conception, and early or recurrent miscarriage. The present study compared the outcomes of ART-based assisted conception in young individuals affected by DOR and young controls with normal ovarian reserve. The findings of this study suggested that, compared to young controls with normal ovarian reserve, those with DOR had reduced clinical pregnancy and live birth rates in fresh ET during IVF/ICSI cycles. Notably, young DOR patients still achieved acceptable clinical pregnancy and live birth rates during IVF/ICSI, and their rates of miscarriage, preterm delivery, and abnormal perinatal outcomes were comparable to those of non-DOR controls. It is widely recognized that age is an essential factor affecting oocyte quality and the clinical pregnancy outcome. Among all the factors affecting ovarian reserve, age is considered the primary factor and an independent predictor of ovarian responsiveness.

The results of this study highlighted that DOR patients needed higher doses of Gn both initially and in total, and exhibited a lowered response to ovulation-promoting drugs. However, this is a retrospective review, the increased dose used for patients in DOR group might be due to subjective bias before starting the treatment. It was also found that young DOR patients, when provided with fresh, high-quality embryos for IVF/ICSI treatment, achieved a clinical pregnancy rate of 47.9% and a live birth rate of 40.6%. These outcomes are consistent with the results of earlier research [13]. The study of Sunkara et al. also indicated a close correlation between the number of oocytes retrieved and the live birth rate of women in IVF treatment, with the highest live birth rate observed when 15 oocytes were retrieved [14]. Moreover, this study demonstrated that DOR did not elevate the risk of miscarriage in young females receiving IVF treatment, which is in line with the findings of Haadsma et al. [15]. The results of another retrospective study, which included 402,185 fresh IVF-ET cycles and 124,351 pregnancy outcomes, showed that the rate of miscarriage after IVF-assisted conception in women was only strongly associated with the woman’s age rather than DOR [16]. In another retrospective study involving 9,489 IVF cycles on females aged 29–44 years, participants were partitioned based on their FSH levels: normal (< 10 IU/L), moderately elevated (10-13.9 IU/L), and highly elevated (14 IU/L). The study findings revealed that women younger than 35 years with DOR did not carry a higher risk of miscarriage, while women with DOR who were of advanced ages were affected to some extent [17]. Thus, these findings suggest that a decrease in ovarian reserve in young women (≤ 35 years) does not necessarily correspond to a decline in oocyte quality. Although diminished follicle numbers and inadequate response to ovarian stimulation are characteristic signs of ovarian aging, younger females with DOR may not exhibit a significant deterioration in oocyte quality due to the effects of age [2].

Birth weight is a crucial factor that influences the health of infants. Infants with lower birth weight are more likely to develop hypertension, diabetes, and other metabolic diseases both in childhood and adulthood compared to infants with normal birth weight [18, 19]. Consequently, the incidence of LBW in infants was explored as a secondary observation in this study. The study results indicated that there was no statistically significant difference in the incidence of LBW at live birth between the DOR and non-DOR groups, and the risk of LBW was comparable in both groups. These findings were consistent with a large retrospective study on IVF treatment, which found no statistically significant difference in the incidence of LBW and very LBW in infants born to women with a low number of oocytes (less than 4 oocytes) compared to those with normal ovarian reserve after confounding factor correction [20]. Similarly, a prior study that classified peak serum estrogen levels during controlled superovulation in individuals undergoing IVF highlighted that the mean birth weight of infants born to patients with low ovarian responsiveness was comparable to that of live births from patients with normal ovarian responsiveness. There was no statistically significant difference in the incidence of LBW and small-for-gestational-age infants [21], which concurs with the findings of this investigation. Moreover, the results of this study also concur with earlier findings that showed no significant difference in birth weight between live-born singleton infants of young women with DOR (≤ 35 years of age) with pregnancy obtained by fresh ET and live-born singleton infants of non-DOR controls of the same age group [5]. Another study, which employed the maximum basal FSH level as an indicator of ovarian reserve, highlighted that the occurrence of LBW in singleton births was not higher among females with DOR than non-DOR controls. Furthermore, the risk of LBW in singleton births was negatively associated with the maximum maternal basal FSH level [22]. Thus, it can be speculated that DOR does not significantly affect the birth weight of offspring born to young females who achieved a live birth through ART.

Meanwhile, this study also evaluated the occurrence of pregnancy complications, including preterm delivery, premature rupture of membranes, gestational hypertension, gestational diabetes, and cesarean delivery between the DOR and non-DOR groups. No statistically significant difference was found between the two groups in this study. These results were consistent with the findings reported by Yang et al. [20], who similarly found that after correcting for confounding factors, the incidence of preterm delivery as well as birth defects in offspring born through IVF did not significantly differ between patients with low ovarian responsiveness and those with normal ovarian responsiveness. In addition, a recent retrospective study indicated that the mean gestational age of offspring born to women with low ovarian responsiveness was not statistically different from that of offspring born alive to patients with normal ovarian responsiveness, and the incidence of preterm births was comparable in both groups [21], which concurs with the results of the current research. In a previous cohort study, the occurrence of hypertensive disorders during pregnancy was used as a primary observation indicator to examine the risk of developing hypertensive disorders during pregnancy among individuals with low ovarian responsiveness who obtained a pregnancy through IVF, and it was found to be similar to that in individuals with normal ovarian responsiveness [22]. Furthermore, the findings of another study highlighted that the occurrence of pregnancy complications and adverse outcomes of live birth singletons after fresh ET-assisted pregnancy in young women with DOR was not higher compared to those of the same age group with normal ovarian reserve [23]. Our current finding concurs with that research. The above findings imply that DOR does not significantly impact the perinatal safety of pregnancy and the health of offspring in young women who obtained live births through IVF. It is also a reasonable conclusion that DOR is not a risk factor for perinatal outcomes in individuals ≤ 35 years who deliver through IVF/ICSI cycles.

This study offers several strengths, including large sample size and comprehensive data. The use of the PSM method to match the two populations based on the selected confounders minimizes the confounding effects and ensures a balance between the two groups. However, it is important to acknowledge certain limitations of this single-center retrospective cohort study. To verify the findings, further prospective studies involving larger sample sizes and multiple centers are necessary to substantiate the findings of this research. In addition, this study specifically focused on young women with DOR undergoing IVF/ICSI in fresh ET cycles. Hence, further studies are required to elucidate the effect of DOR on the outcomes of young females undergoing frozen-thaw ET cycles.

In summary, this study confirms that DOR lowers clinical pregnancy and live birth rates in young females undergoing fresh ET in IVF/ICSI cycles. However, it also establishes that the risk of perinatal complications in females with DOR aged ≤ 35 years undergoing IVF/ICSI are comparable to those in the non-DOR group. These findings contribute to the evidence-based understanding of the treatment of DOR patients and suggest that young women with DOR can still achieve favorable pregnancy outcomes once they have high quality embryos for transfer, with no increased risk of adverse perinatal and neonatal outcomes. Such knowledge can facilitate the confidence of young women with DOR in pursuing assisted reproduction techniques to have healthy children.

## Data Availability

The datasets used and/or analyzed during the current study are available from the corresponding authors on reasonable request.
